# IgA nephropathy in a child with X-linked agammaglobulinemia: a case report

**DOI:** 10.1186/s12887-024-04746-7

**Published:** 2024-04-30

**Authors:** Yuanjin Song, Lili Sun, Dongning Feng, Qing Sun, Yibing Wang

**Affiliations:** Department of Nephrology and Immunology, Qingdao Women and Children’s Hospital, Qingdao, China

**Keywords:** X-linked agammaglobulinemia, IgA nephropathy, Hematuria, Proteinuria, Case report

## Abstract

**Background:**

X-linked agammaglobulinemia (XLA) is a primary immunodeficiency disease caused by mutations in the Bruton tyrosine kinase (BTK) gene. Individuals diagnosed with XLA are at an increased risk of developing autoimmune diseases. However, renal involvement are rare in cases of XLA.

**Case presentation:**

In this report, we discussed a specific case involving a 6-year-old boy with XLA who experienced recurrent upper respiratory tract infections since the age of one. He presented with symptoms of hematuria and proteinuria, and renal pathology confirmed the presence of immunoglobulin (Ig) A nephropathy. Treatment comprised glucocorticoids, mycophenolate mofetil, and intermittent intravenous immunoglobulin replacement therapy. Consequently, there was a remission of proteinuria and a partial improvement in hematuria.

**Conclusions:**

In this study, we describe the first case of IgA nephropathy associated with XLA. This is an interesting phenotype found in XLA, and it provides valuable insights into the process of autoimmunity and the regulation of immune function in individuals with XLA. Based on our findings, we recommend the evaluation of immunoglobulin levels in patients diagnosed with IgA nephropathy.

## Background

X-linked agammaglobulinemia (XLA) is a primary immunodeficiency disease characterized by the lack of peripheral B cells and low levels of serum immunoglobulins, predisposing individuals to recurring infections [1]. The underlying etiology of XLA is attributed to mutations in the Bruton tyrosine kinase (BTK) gene, which is situated on the long arm of the X-chromosome [1]. BTK plays a crucial role in regulating various signaling pathways and is essential for the differentiation and maturation of immature B-lymphocytes [1, 2].  Up to year 2023, more than 2,300 variants have been identified in BTK gene.

Despite XLA patients exhibiting low serum immunoglobulin concentrations and defective antibody responses, they have an increased risk of developing autoimmune diseases such as juvenile idiopathic arthritis, juvenile dermatomyositis, inflammatory bowel disease, and Kawasaki disease [3, 4]. Renal involvement in XLA is rarely observed, and previous studies have reported instances of membranoproliferative glomerulonephritis (MPGN) and membranous glomerulopathy (MG) in XLA patients receiving intravenous immunoglobulin (IVIG) therapy [5–7]. In this present study, we describe the first case of immunoglobulin (Ig) A nephropathy associated with XLA.

## Case presentation

In June 2022, a six-year-old boy was admitted to our hospital, presenting with the primary complaint of macroscopic hematuria persisting for one month following an episode of acute laryngitis. The child had been experiencing recurrent upper respiratory tract infections since the age of one, with no documented history of major infections during his early childhood. He was born to nonconsanguineous parents at 39 weeks’ gestation. The medical family history was unremarkable. Physical examination showed no positive indications such as elevated blood pressure or edema in the bilateral lower limbs. The child’s growth and psychomotor development were normal.

Blood routine test suggested mild anemia (hemoglobin 102 g/L). Urinalysis indicated the presence of 968.6 erythrocytes per high power field (HPF), 7.81 white blood cells per HPF, and proteinuria graded at 1 + . 24-h urine protein determination revealed excretion of 454.18 mg (18.75 mg/kg) of protein (normal, < 150 mg/24 h). Renal function was within the normal range with a urea nitrogen of 6.87 mmol/L and serum creatinine of 37.13 umol/L. The evaluations for serum electrolytes, liver function, C-reactive protein, antistreptolysin O, coagulation profile, and thyroid function tests were all within the normal ranges and HIV, HBV, and HCV infections were excluded. Tests for anti-neutrophil cytoplasmic antibodies, anti-nuclear antibodies and anti-double-stranded DNA antibodies were negative. Immunologic evaluation showed low but detectable IgG level of 3.24 g/L (reference range, 5.53–13.07), IgM < 0.17 g/L (reference range, 0.56–2.18), IgA 1.57 g/L (reference range, 0.23–1.98) and IgE 344.8 IU/ml (reference range, 0–90). Low serum complement levels were observed, with C3 level of 0.35 g/L (reference range 0.88–2.01) and C4 of 0.26 g/L (reference range 0.16–0.47). Additional evaluation revealed natural killer cell and T-lymphocyte counts were normal, with 1.15% of CD19 + B cells.

**Fig. 1:**
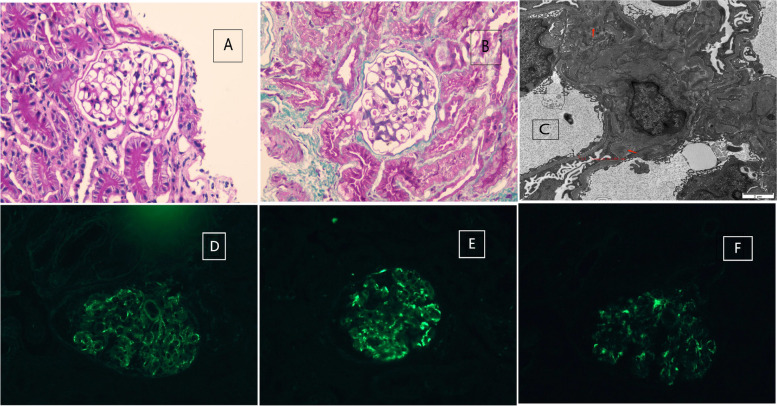
Histology of the patient's renal biopsy. **A** and **B** Light microscopy showing mild focal segmental proliferation of glomerular mesangial cells in PAS stain (**A**) and in MASSON stain (**B**). **C** Electron microscopy exhibiting numerous electron-dense deposits in the mesangium(red arrows). **D**, **E** and **F** Immunofluorescence showing deposition of IgA (+ + +) (**D**), C3(+ +) (**E**) and IgM( +) (**F**) in the mesangial area. PAS, Periodic acid-Schiff

The ultrasonography results of the kidney were normal. Results of renal biopsy demonstrated mild focal segmental proliferation of glomerular mesangial cells and the matrix in the 14 glomeruli obtained by light microscopy. Segmental sclerosis and crescent formation were absent. Renal tubular epithelial cells displayed vacuolar and granular degeneration, with no obvious atrophy. Arteries and arterioles were unremarkable. Two glomeruli were detected under electron microscopy. There was no obvious endothelial cell proliferation. The thickness of the basement membrane was about 180-350 nm. Partially fusion of foot processes was also observed. The mesangial cells and matrix proliferated, and electron-dense deposits were noted in the mesangial area. Immunofluorescent analysis revealed a strong IgA granular deposition (+ + +) in the mesangial area, along with mesangial staining for C3 (+ +) and IgM( +). Based on these results, the patient was diagnosed with IgA nephropathy (Fig. 1).

**Fig. 2:**
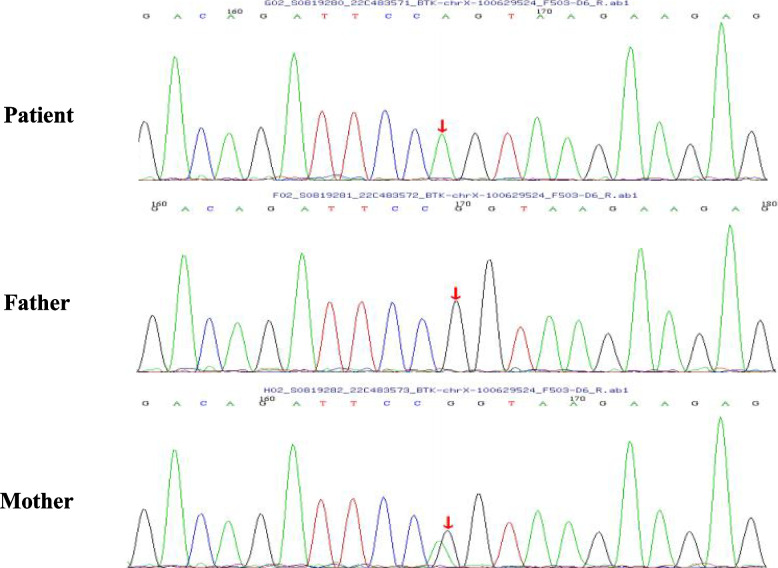
Genetic analysis revealed a hemizygous c.240G > A mutation in the BTK gene of the patient and a heterozygous c.240G > A mutation in the BTK gene of his mother

Given the observed hypogammaglobulinemia, the deficiency of CD19 + B-lymphocytes, and the patient’s male gender, a diagnosis of X-Linked Agammaglobulinemia (XLA) was considered. Blood samples were obtained from the proband and his parents for genetic analysis. DNA was extracted using the Blood Genome Column Medium Extraction Kit (Kangweishiji, China) and subjected to exome enrichment with the xGen Exome Research Panel v2.0(IDT, Iowa, USA). Sequencing was performed on the DNBSEQ-T7 platform (MGI, China), with data preprocessing to eliminate adapters and low-quality reads. Alignment to the GRCh37/hg19 reference genome was done using BWA, followed by variant calling with GATK and annotation via ANNOVAR. Variant analysis utilized computational tools like SIFT, Polyphen-2, and MutationTaster, alongside splice site prediction with MaxEntScan and SpliceAI, focusing on rare variants within genes relevant to the phenotype. Trio analysis was applied to assess inheritance patterns, with candidate variants manually reviewed and validated by Sanger sequencing according to American College of Medical Genetics and Genomics (ACMG) guidelines. This process identified a hemizygous variant in the BTK gene (NM_000061.3), c.240G > A (p.Pro80 =). Although the variant is synonymous, it occurs at the penultimate base of exon 3, which can potentially affect mRNA splicing, leading to a significantly altered protein sequence (Fig. 2).

He was diagnosed with XLA and IgA nephropathy. His treatment included pulse methylprednisolone at a daily dosage of 10 mg/kg for three days, subsequently reduced to 1 mg/kg/day and gradually tapered. Additionally, mycophenolate mofetil was initiated at a daily dosage of 20 mg/kg. The treatment also included monthly IVIG therapy. However, due to economic constraints, the patient had poor compliance with IVIG treatment. Fortunately, he did not experience any major episodes of infections. One year and three months post-discharge, the latest urinalysis during his follow-up indicated a negative result for urine protein and revealed 14.3 erythrocytes /HPF. His proteinuria was resolved and hematuria was partially improved.

## Discussion and conclusions

To the best of our knowledge, this is the first case report of IgA nephropathy in an XLA patient. It was characterized by recurrent upper respiratory tract infections, hypogammaglobulinemia, and IgA nephropathy manifesting with symptoms of gross hematuria and proteinuria. The patient received treatment with glucocorticoids, mycophenolate mofetil, and intermittent intravenous immunoglobulin replacement therapy. At follow-up, there was a resolution of proteinuria and a partial improvement in hematuria.

Our patient presents with an atypical form of XLA, characterized by a reduced number of B cells and low IgM levels, alongside low yet detectable IgG levels and normal IgA. Previous research has identified cases of atypical XLA in which patients display concentrations of one or more specific immunoglobulin isotypes that are normal or nearly normal [8–10]. The underlying cause of this phenotype remains unclear. Specific mutations in the BTK gene impacting XLA might result in partial preservation of BTK enzyme functionality, leading to detectable circulating B cells, elevated immunoglobulin levels, and milder clinical manifestations [8, 11]. In individuals presenting with atypical XLA,the employment of genetic analysis facilitates an accurate diagnosis.

Multiple investigations have showed that IgA nephropathy is classified as an autoimmune disease [12]. This is due to the production and recognition of Gd-IgA1 as an autoantigen by circulating antiglycan autoantibodies,thus resulting in the formation of nephritogenic immune complexes [12, 13]. It is worth noting that individuals with deficiencies in CD19 and CD81 have been observed to develop IgA nephropathy [14, 15]. Besides, increased serum levels of Gd-IgA1 and circulating immune complexes containing IgA and IgG were also observed in Wiskott-Aldrich syndrome (WAS) complicating with IgA nephropathy [16]. In our XLA patient, IgA and complement deposits were demonstrated in mesangial areas and complement may have been activated locally after immune complex formation. This process could be attributed to the dysfunction of the peripheral B-lymphocytes and interactions between endogenous residual IgG and an endogenous antigen [3, 17].

There were few reports of renal involvement in XLA. Previous studies have described cases of XLA patients developing conditions such as membranous nephropathy, membranoproliferative glomerulonephritis, lupus-like nephritis and tubulointerstitial nephritis while undergoing IVIG treatment, which were immune complex-mediated disorders with evidence of complement activation [5–8, 18, 19]. However, Jiao Chenfeng et al. reported a case of a teenage boy with XLA who developed membranoproliferative glomerulonephritis, even though he did not receive IVIG infusion [20]. Interestingly, our case involved a boy who also did not undergo immunoglobulin replacement therapy. This suggests that, in our case, nephropathy could be considered as a manifestation of autoimmune phenomena associated with XLA. Recurrent or subclinical infections observed in XLA patients could lead to chronic inflammation, immune dysregulation, and the production of autoantibodies. Such processes are likely to foster autoimmune mechanisms that contribute to the emergence of IgA nephropathy [21, 22]. Additionally, excessive stimulation by pathogen molecules of Toll-like receptors (TLRs) may contribute to inducing autoimmunity in XLA [21, 22]. Further investigation is needed to more comprehensively understand the underlying mechanisms.

In this study, we have presented an uncommon and interesting autoimmune phenomenon, IgA nephropathy, in a patient with XLA. Although the exact pathogenesis is unclear, it highlights the importance of understanding the development of autoimmunity and regulation of immune function in patients with XLA. Additionally, based on the findings, we advocate for the routine assessment of immunoglobulin levels in patients diagnosed with IgA nephropathy, especially those with a history of recurrent respiratory infections. This could facilitate early intervention and possibly enhance clinical outcomes.

## Data Availability

The genetic data used in our study are publicly available on LOVD (https://databases.lovd.nl/shared/variants/0000971535#00000685). Additionally, these data are available in the ClinVar database (https://www.ncbi.nlm.nih.gov/clinvar/variation/632730/?oq).

## Abbreviations

XLA: X-linked agammaglobulinemia
BTK: Bruton tyrosine kinase
MPGN: Membranoproliferative glomerulonephritis
MG: Membranous glomerulopathy
IVIG: Intravenous immunoglobulin
Ig: Immunoglobulin
HPF: High power field
WES: Whole exome sequencing
ACMG: American College of Medical Genetics and Genomics
WAS: Wiskott-Aldrich syndrome
TLRs: Toll-like receptors

## References

[1] El-Sayed ZA, Abramova I, Aldave JC, Al-Herz W, Bezrodnik L, Boukari R (2019). X-linked agammaglobulinemia (XLA): phenotype, diagnosis, and therapeutic challenges around the world. World Allergy Organ J.

[2] Weber ANR, Bittner Z, Liu X, Dang T-M, Radsak MP, Brunner C (2017). Bruton's tyrosine kinase: an emerging key player in innate immunity. Front Immunol.

[3] Hernandez-Trujillo VP, Scalchunes C, Cunningham-Rundles C, Ochs HD, Bonilla FA, Paris K (2014). Autoimmunity and inflammation in X-linked agammaglobulinemia. J Clin Immunol.

[4] Rivas-Larrauri F, Aguilar-Zanela L, Castro-Oteo P, Rosales-Hernandez LA, Otero-Mendoza F, López-Herrera G (2019). Kawasaki disease and immunodeficiencies in children: case reports and literature review. Rheumatol Int.

[5] Yoshino A, Honda M, Kanegane H, Obata K, Matsukura H, Sakazume S (2006). Membranoproliferative glomerulonephritis in a patient with X-linked agammaglobulinemia. Pediatr Nephrol.

[6] Endo LM, Giannobile JV, Dobbs AK, Foote JB, Szymanska E, Warnock DG (2011). Membranous glomerulopathy in an adult patient with X-linked agammaglobulinemia receiving intravenous gammaglobulin. J Investig Allergol Clin Immunol.

[7] Lavrador V, Correia F, Sampaio R, Cândido C, Sameiro-Faria M, Marques L (2014). Membranoproliferative glomerulonephritis and x-linked agammaglobulinemia: an uncommon association. Case Rep Pediatr.

[8] Lim LM, Chang JM, Wang IF, Chang WC, Hwang DY, Chen HC (2013). Atypical X-linked agammaglobulinaemia caused by a novel BTK mutation in a selective immunoglobulin M deficiency patient. BMC Pediatr.

[9] Chear CT, Ismail IH, Chan KC, Noh LM, Kassim A, Latiff AH (2023). Clinical features and mutational analysis of X-linked agammaglobulinemia patients in Malaysia. Front Immunol.

[10] Han SP, Lin YF, Weng HY, Tsai SF, Fu LS (2019). A novel BTK gene mutation in a child with atypical x-linked agammaglobulinemia and recurrent hemophagocytosis: a case report. Front Immunol.

[11] Broides A, Yang W, Conley ME (2006). Genotype/phenotype correlations in X-linked agammaglobulinemia. Clin Immunol.

[12] Lafayette RA, Kelepouris E (2018). Immunoglobulin a nephropathy: advances in understanding of pathogenesis and treatment. Am J Nephrol.

[13] Lavrador V, Correia F, Sampaio R, Cândido C, Sameiro-Faria M, Marques L (2016). IgA nephropathy. Nat Rev Dis Primers.

[14] Yang Lu, Liu P, Hongqiang Du, Chen R, Zhou Bo, Li Y (2022). Novel CD81 mutations in a chinese patient led to IgA nephropathy and impaired BCR signaling. J Clin Immunol.

[15] Vince N, Boutboul D, Mouillot G, Just N, Peralta M, Casanova J-L (2011). Defects in the CD19 complex predispose to glomerulonephritis, as well as IgG1 subclass deficiency. J Allergy Clin Immunol.

[16] Liu C-H, Kang-Hsi Wu, Lin T-Y, Wei C-C, Lin C-Y, Chen X-X (2013). Wiskott-Aldrich syndrome with IgA nephropathy: a case report and literature review. Int Urol Nephrol.

[17] Ng Y-S, Wardemann H, Chelnis J, Cunningham-Rundles C, Meffre E (2004). Bruton's tyrosine kinase is essential for human B cell tolerance. J ExpMed.

[18] Sugimoto K, Nishi H, Miyazawa T, Wada N, Izu A, Enya T (2014). Tubulointerstitial nephritis complicating IVIG therapy for X-linked agammaglobulinemia. BMC Nephrol.

[19] Takeguchi M, Korematsu S, Miyahara H, Kuga S, Izumi T (2017). IVIG-triggered tubulointerstitial nephritis in X-linked agammaglobulinemia. Pediatr Int.

[20] Chenfeng JIAO, Lili ZHAO, Ling JIANG, Feng XU, Zhen CHENG (2021). X-linked agammaglobulinemia with membranoproliferative glomerulonephritis. J Nephrol Daily Transplant.

[21] Knight AK, Cunningham-Rundles C (2006). Inflammatory and autoimmune complications of common variable immune deficiency. Autoimmun Rev.

[22] Kubo T, Uchida Y, Watanabe Y, Abe M, Nakamura A, Ono M (2009). Augmented TLR9-induced Btk activation in PIR-B-deficient B-1 cells provokes excessive autoantibody production and autoimmunity. J Exp Med.

